# Supplementary material for: The Small GTPase Arf6 Functions as a Membrane Tether in a Chemically-Defined Reconstitution System
Source: Front Cell Dev Biol. 2021 Jan 28;9:628910. doi: 10.3389/fcell.2021.628910 (PMC7876375; doi:10.3389/fcell.2021.628910)
Supplement: Supplementary file 1 [file Data_Sheet_1.PDF]

Supplemental Figures

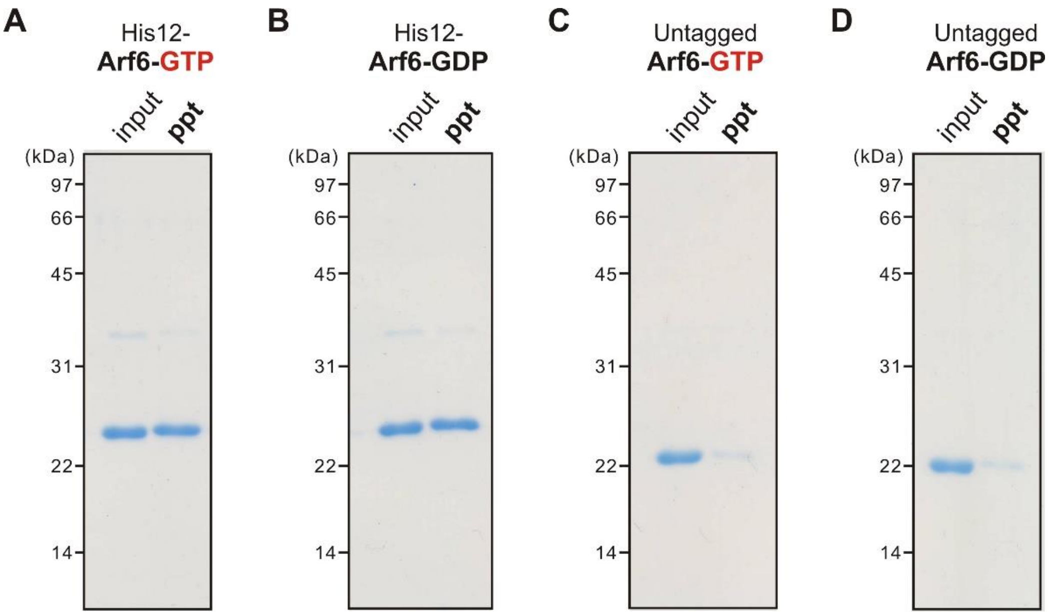

Figure S1. Liposome co-sedimentation assays testing the membrane association of nucleotide-loaded Arf6 proteins. (**A**-**D**) Purified nucleotide-loaded Arf6 proteins (4 μM), including His12-Arf6-GTP (**A**), His12-Arf6-GDP (**B**), untagged Arf6-GTP lacking a His12 tag (**C**), and untagged Arf6-GDP (**D**), were incubated with DOGS-NTA-bearing liposomes (2 mM lipids; 1,000-nm diameter) in RB150 containing 5 mM $MgCl_2$ and 1 mM DTT (30°C, 30 min), centrifuged (20,000 × *g*, 30 min, 4°C), and analyzed by SDS-PAGE and CBB staining for precipitates (*ppt*) obtained after centrifugation.

Supplemental Figures

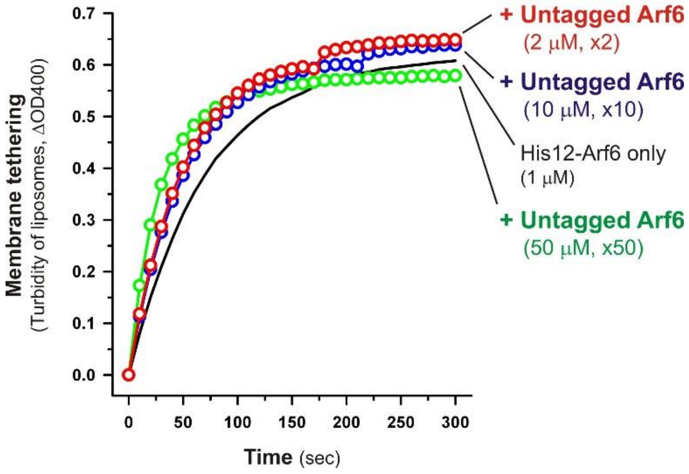

Figure S2. Selective *trans*-assembly between membrane-anchored Arf6 proteins in reconstituted membrane tethering. Purified His12-Arf6 proteins (1 μM) were mixed with excess amounts of untagged Arf6 proteins lacking a His12-tag membrane anchor (2 μM, 10 μM, or 50 μM), incubated (30°C, 10 min), supplemented with DOGS-NTA-bearing liposomes (1 mM lipids; 200-nm diameter), and subjected to liposome turbidity assays, as in **Figure 2B**.

Supplemental Figures

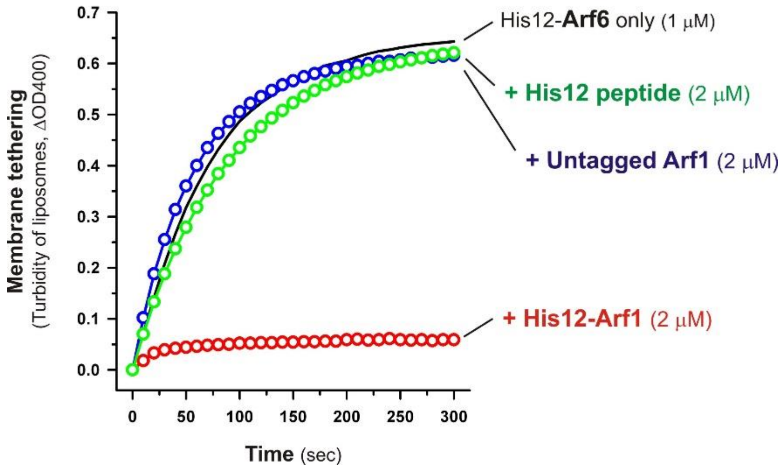

Figure S3. Specific inhibitory effect of membrane-anchored Arf1 on Arf6-mediated membrane tethering. His12-Arf6 (1 μM) was mixed with His6-Arf1 (2 μM), untagged Arf1 lacking a His12 tag (2 μM), or a His12 peptide (2 μM), incubated (30°C, 10 min), mixed with DOGS-NTA-bearing liposomes (1 mM lipids; 200-nm diameter), and subjected to liposome turbidity assays, as in **Figure 4B**.
